# 1,5-Dibromo-2,4-dimeth­oxy­benzene

**DOI:** 10.1107/S1600536812047848

**Published:** 2012-11-30

**Authors:** A. M. Vijesh, Arun M. Isloor, Thomas Gerber, Benjamin van Brecht, Richard Betz

**Affiliations:** a GITAM University, Department of Engineering Chemistry, GIT, Rushikonda, Visakhapatnam, A.P. 530 045, India; b National Institute of Technology-Karnataka, Department of Chemistry, Medicinal Chemistry Laboratory, Surathkal, Mangalore 575 025, India; c Nelson Mandela Metropolitan University, Summerstrand Campus, Department of Chemistry, University Way, Summerstrand, PO Box 77000, Port Elizabeth, 6031, South Africa

## Abstract

In the title compound, C_8_H_8_Br_2_O_2_, all non-H atoms lie essentially in a common plane (r.m.s deviation of all fitted non-H atoms = 0.0330 Å). In the crystal, weak C—H⋯O hydrogen bonds connect the mol­ecules, forming chains which extend along the *b*-axis direction.

## Related literature

For background to the pharmacological importance of the title compound, see: Pahari & Rohr (2009 ▶). For the synthesis of the title compound, see: Yang *et al.* (2009 ▶). For a report listing the crystal structure of 1-bromo-5-chloro-2,4-dimeth­oxy­benzene but entered incorrectly as the title compound in the CSD (TASBAR), see: Yang *et al.* (2005 ▶). For graph-set analysis of hydrogen bonds, see: Bernstein *et al.* (1995 ▶).
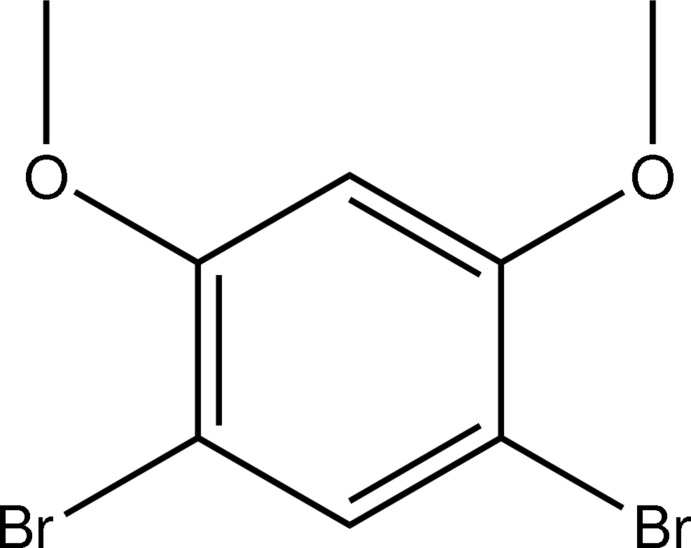


## Experimental

### 

#### Crystal data

C_8_H_8_Br_2_O_2_
*M* *_r_* = 295.96
Monoclinic, 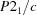

*a* = 7.7944 (2) Å
*b* = 8.5884 (4) Å
*c* = 14.7877 (4) Å
β = 107.838 (1)°
*V* = 942.32 (6) Å^3^
*Z* = 4
Mo *K*α radiation
μ = 8.56 mm^−1^
*T* = 200 K
0.47 × 0.46 × 0.34 mm

#### Data collection

Bruker APEXII CCD diffractometer
Absorption correction: multi-scan (*SADABS*; Bruker, 2008 ▶) *T*_min_ = 0.674, *T*_max_ = 1.000
15097 measured reflections
2350 independent reflections
2094 reflections with *I* > 2σ(*I*)
*R*_int_ = 0.032

#### Refinement

*R*[*F* ^2^ > 2σ(*F* ^2^)] = 0.021
*wR*(*F* ^2^) = 0.049
*S* = 1.08
2350 reflections
111 parameters
H-atom parameters constrained
Δρ_max_ = 0.55 e Å^−3^
Δρ_min_ = −0.52 e Å^−3^

### 

Data collection: *APEX2* (Bruker, 2010 ▶); cell refinement: *SAINT* (Bruker, 2010 ▶); data reduction: *SAINT*; program(s) used to solve structure: *SHELXS97* (Sheldrick, 2008 ▶); program(s) used to refine structure: *SHELXL97* (Sheldrick, 2008 ▶); molecular graphics: *ORTEP-3* (Farrugia, 2012 ▶) and *Mercury* (Macrae *et al.*, 2008 ▶); software used to prepare material for publication: *SHELXL97* and *PLATON* (Spek, 2009 ▶).

## Supplementary Material

Crystal structure: contains datablock(s) I, global. DOI: 10.1107/S1600536812047848/zs2243sup1.cif

Supplementary material file. DOI: 10.1107/S1600536812047848/zs2243Isup2.cdx

Structure factors: contains datablock(s) I. DOI: 10.1107/S1600536812047848/zs2243Isup3.hkl

Supplementary material file. DOI: 10.1107/S1600536812047848/zs2243Isup4.cml

Additional supplementary materials:  crystallographic information; 3D view; checkCIF report

## Figures and Tables

**Table 1:** Hydrogen-bond geometry (Å, °)

*D*—H⋯*A*	*D*—H	H⋯*A*	*D*⋯*A*	*D*—H⋯*A*
C7—H7*C*⋯O1^i^	0.98	2.70	3.632 (3)	160

Symmetry code: (i) 
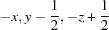
.

**Fig. 1.**
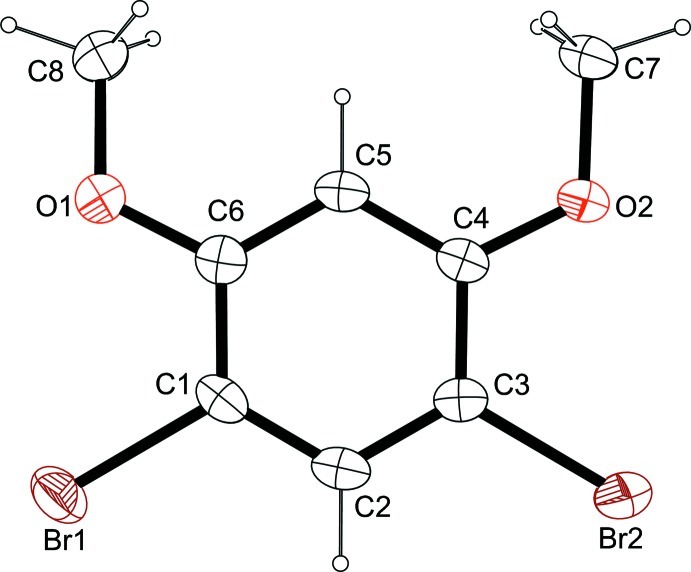
The molecular structure of the title compound showing atom labels, with anisotropic displacement ellipsoids drawn at the 50% probability level.

**Fig. 2.**
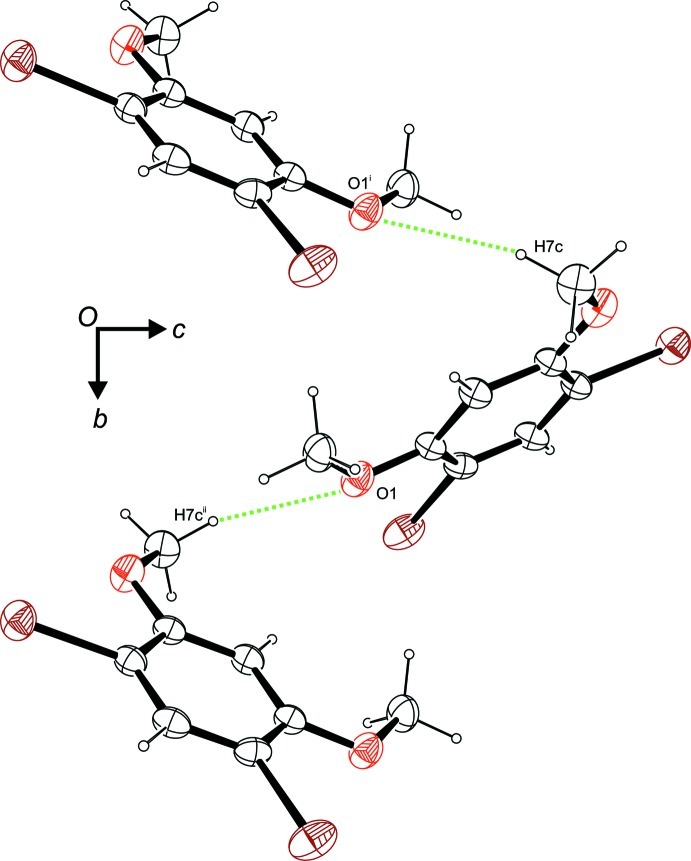
Intermolecular contacts, viewed along [-1 0 0], with hydrogen bonds shown as dashed lines. For symmetry codes, see Table 1.

**Fig. 3.**
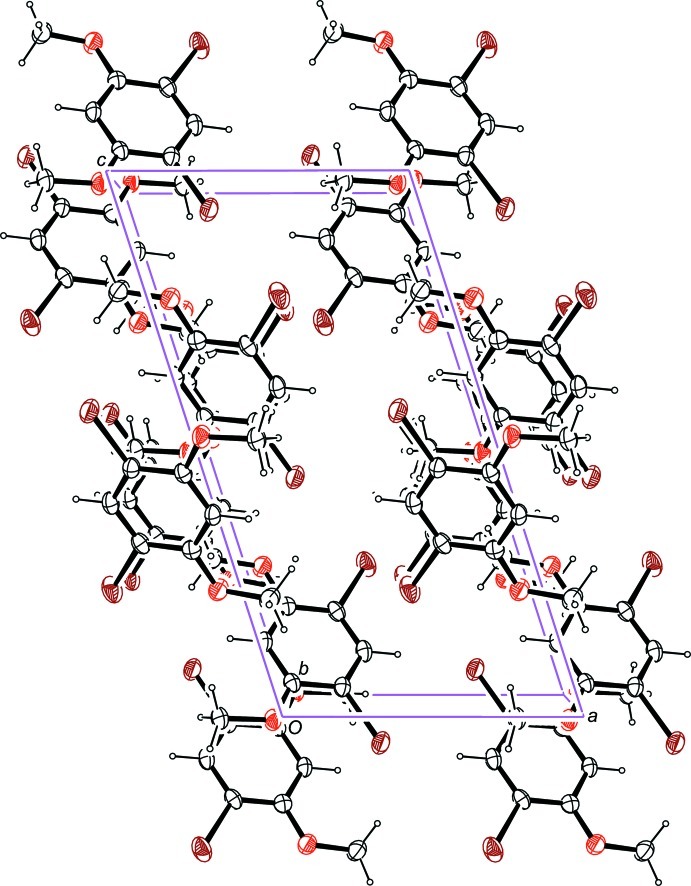
Molecular packing of the title compound, viewed down [0 1 0] (anisotropic displacement ellipsoids drawn at 50% probability level).

## supplementary crystallographic information

### Comment

The title compound 1,5-dibromo-2,4-dimethoxybenzene is an important intermediate for the synthesis of the anti-HIV drug Elvitegravir and the anticancer drug Psoralidin (Pahari *et al.*, 2009). The crystal structure of 1-bromo-5-chloro-2,4-dimethoxybenzene has been determined (Yang *et al.*, 2005), but the compound was in fact reported erroneously as 1,5-dibromo-2,4-dimethoxybenzene in the CSD (CCDC 271922, ref-code: TASBAR). The error is the result of 50% rotational disorder between the two halogen atoms in the molecule and this fact is also mentioned among the deposited experimental details in the CSD entry. Furthermore, the cell constants reported in this entry [*a* = 7.722 (4) Å, *b* = 7.949 (3) Å, *c* = 7.405 (3) Å, β = 91.78 (1) °, *V* = 454.315 Å^3^, *Z* = 2, space group *P* 2/*c*] differed significantly from those of the title compound. In view of the importance of the title compound in synthetic as well as medicinal chemistry, and to rectify the anomaly in the deposited crystallographic data, it was resynthesized and the crystal structure is reported herein.

The title compound is essentially planar (r.m.s. of all fitted non-hydrogen atoms = 0.0330 Å), with one of the methyl carbon atoms deviating most from this common plane by -0.073 (2) Å (Fig. 1). Intracyclic C–C–C angles cover a range from 118.96 (16) ° to 120.73 (17)° with the smallest angle found at one of the carbon atoms bearing a methoxy substituent (C4) and the largest angle at the bromo-substituted carbon atom (C1) in the *para* position to C4.

In the crystal, a weak intermolecular methyl C—H···O contact (Table 1) whose value falls slightly below the sum of van-der-Waals radii of the atoms participating, is observed, connecting the molecules into chains which extend along *b* (Fig. 2). A C1–Br1···*Cg*^ii^ contact is also present. According to a graph-set analysis (Bernstein *et al.*, 1995), the descriptor for the C–H···O contacts is *C*(7). The shortest intercentroid distance between two aromatic ring systems is 4.0267 (10) Å (Fig. 3).

### Experimental

1,5-Dibromo-2,4-dimethoxybenzene was synthesized according to a published procedure (Yang *et al.*, 2009). The crude product was recrystallized from hot ethanol.

### Refinement

Carbon-bound H atoms were placed in calculated positions [C—H(aromatic) = 0.95 Å and C—H(methyl) = 0.98 Å] and with *U*_iso_(H) = 1.2*U*_eq_(C) (aromatic) and allowed to ride in the refinement. The H atoms of the methyl groups were allowed to rotate with a fixed angle around the C—C bond to best fit the experimental electron density, with *U*_iso_(H) = 1.5*U*_eq_(C).

### Crystal data

**Table:**

C_8_H_8_Br_2_O_2_	*F*(000) = 568
*M**_r_* = 295.96	*D*_x_ = 2.086 Mg m^−^^3^
Monoclinic, *P*2_1_/*c*	Melting point = 414–413 K
Hall symbol: -P 2ybc	Mo *K*α radiation, λ = 0.71073 Å
*a* = 7.7944 (2) Å	Cell parameters from 9980 reflections
*b* = 8.5884 (4) Å	θ = 2.7–28.3°
*c* = 14.7877 (4) Å	µ = 8.56 mm^−^^1^
β = 107.838 (1)°	*T* = 200 K
*V* = 942.32 (6) Å^3^	Block, white
*Z* = 4	0.47 × 0.46 × 0.34 mm

### Data collection

**Table:**

Bruker APEXII CCD diffractometer	2350 independent reflections
Radiation source: fine-focus sealed tube	2094 reflections with *I* > 2σ(*I*)
Graphite monochromator	*R*_int_ = 0.032
φ and ω scans	θ_max_ = 28.4°, θ_min_ = 2.8°
Absorption correction: multi-scan (*SADABS*; Bruker, 2008)	*h* = −10→10
*T*_min_ = 0.674, *T*_max_ = 1.000	*k* = −11→11
15097 measured reflections	*l* = −18→19

### Refinement

**Table:**

Refinement on *F*^2^	Primary atom site location: structure-invariant direct methods
Least-squares matrix: full	Secondary atom site location: difference Fourier map
*R*[*F*^2^ > 2σ(*F*^2^)] = 0.021	Hydrogen site location: inferred from neighbouring sites
*wR*(*F*^2^) = 0.049	H-atom parameters constrained
*S* = 1.08	*w* = 1/[σ^2^(*F*_o_^2^) + (0.0199*P*)^2^ + 0.5315*P*] where *P* = (*F*_o_^2^ + 2*F*_c_^2^)/3
2350 reflections	(Δ/σ)_max_ = 0.001
111 parameters	Δρ_max_ = 0.55 e Å^−^^3^
0 restraints	Δρ_min_ = −0.52 e Å^−^^3^

### Fractional atomic coordinates and isotropic or equivalent isotropic displacement parameters (Å^2^)

**Table:**

	*x*	*y*	*z*	*U*_iso_*/*U*_eq_	
Br1	0.43964 (3)	0.63810 (3)	0.268745 (16)	0.04029 (7)	
Br2	0.29505 (3)	0.26527 (3)	−0.060701 (15)	0.03453 (7)	
O1	0.07656 (18)	0.52208 (17)	0.26640 (10)	0.0321 (3)	
O2	−0.04398 (17)	0.19995 (17)	−0.01445 (10)	0.0323 (3)	
C1	0.2839 (2)	0.5013 (2)	0.18080 (13)	0.0256 (4)	
C2	0.3359 (2)	0.4436 (2)	0.10619 (13)	0.0265 (4)	
H2	0.4487	0.4729	0.0992	0.032*	
C3	0.2236 (2)	0.3432 (2)	0.04159 (13)	0.0243 (4)	
C4	0.0576 (2)	0.2998 (2)	0.05093 (13)	0.0235 (4)	
C5	0.0069 (2)	0.3587 (2)	0.12664 (13)	0.0250 (4)	
H5	−0.1056	0.3292	0.1339	0.030*	
C6	0.1190 (2)	0.4601 (2)	0.19174 (13)	0.0244 (4)	
C7	−0.2196 (3)	0.1625 (3)	−0.00979 (15)	0.0338 (4)	
H7A	−0.2815	0.0957	−0.0636	0.051*	
H7B	−0.2888	0.2585	−0.0123	0.051*	
H7C	−0.2089	0.1075	0.0498	0.051*	
C8	−0.0860 (3)	0.4691 (3)	0.28213 (15)	0.0340 (4)	
H8A	−0.0983	0.5179	0.3397	0.051*	
H8B	−0.0816	0.3557	0.2898	0.051*	
H8C	−0.1894	0.4976	0.2277	0.051*	

### Atomic displacement parameters (Å^2^)

**Table:**

	*U*^11^	*U*^22^	*U*^33^	*U*^12^	*U*^13^	*U*^23^
Br1	0.02623 (10)	0.04018 (13)	0.05077 (14)	−0.00833 (8)	0.00632 (9)	−0.01578 (9)
Br2	0.02932 (11)	0.04093 (12)	0.03863 (12)	−0.00448 (8)	0.01823 (9)	−0.00465 (8)
O1	0.0301 (7)	0.0358 (8)	0.0333 (7)	−0.0062 (6)	0.0138 (6)	−0.0083 (6)
O2	0.0238 (7)	0.0400 (8)	0.0353 (7)	−0.0105 (6)	0.0123 (6)	−0.0101 (6)
C1	0.0209 (8)	0.0209 (8)	0.0314 (9)	−0.0031 (6)	0.0026 (7)	−0.0002 (7)
C2	0.0195 (8)	0.0241 (9)	0.0356 (10)	−0.0015 (7)	0.0082 (7)	0.0049 (7)
C3	0.0215 (8)	0.0235 (9)	0.0288 (9)	0.0008 (6)	0.0093 (7)	0.0039 (7)
C4	0.0197 (8)	0.0222 (8)	0.0272 (9)	−0.0012 (7)	0.0053 (7)	0.0030 (7)
C5	0.0195 (8)	0.0257 (9)	0.0309 (9)	−0.0025 (7)	0.0094 (7)	0.0026 (7)
C6	0.0248 (8)	0.0218 (8)	0.0263 (9)	0.0015 (7)	0.0071 (7)	0.0034 (7)
C7	0.0227 (9)	0.0418 (11)	0.0371 (11)	−0.0098 (8)	0.0093 (8)	−0.0034 (9)
C8	0.0336 (10)	0.0397 (11)	0.0328 (10)	−0.0050 (8)	0.0165 (8)	−0.0030 (9)

### Geometric parameters (Å, º)

**Table:**

Br1—C1	1.8918 (18)	C3—C4	1.393 (2)
Br2—C3	1.8873 (19)	C4—C5	1.392 (3)
O1—C6	1.355 (2)	C5—C6	1.391 (3)
O1—C8	1.431 (2)	C5—H5	0.9500
O2—C4	1.352 (2)	C7—H7A	0.9800
O2—C7	1.428 (2)	C7—H7B	0.9800
C1—C2	1.379 (3)	C7—H7C	0.9800
C1—C6	1.389 (2)	C8—H8A	0.9800
C2—C3	1.382 (3)	C8—H8B	0.9800
C2—H2	0.9500	C8—H8C	0.9800
			
C6—O1—C8	117.21 (15)	C4—C5—H5	119.6
C4—O2—C7	117.93 (15)	O1—C6—C1	117.41 (16)
C2—C1—C6	120.73 (17)	O1—C6—C5	123.47 (16)
C2—C1—Br1	119.28 (13)	C1—C6—C5	119.12 (17)
C6—C1—Br1	119.99 (14)	O2—C7—H7A	109.5
C1—C2—C3	119.87 (17)	O2—C7—H7B	109.5
C1—C2—H2	120.1	H7A—C7—H7B	109.5
C3—C2—H2	120.1	O2—C7—H7C	109.5
C2—C3—C4	120.59 (17)	H7A—C7—H7C	109.5
C2—C3—Br2	119.71 (13)	H7B—C7—H7C	109.5
C4—C3—Br2	119.70 (14)	O1—C8—H8A	109.5
O2—C4—C5	123.84 (16)	O1—C8—H8B	109.5
O2—C4—C3	117.20 (16)	H8A—C8—H8B	109.5
C5—C4—C3	118.96 (16)	O1—C8—H8C	109.5
C6—C5—C4	120.72 (16)	H8A—C8—H8C	109.5
C6—C5—H5	119.6	H8B—C8—H8C	109.5
			
C6—C1—C2—C3	−0.3 (3)	O2—C4—C5—C6	179.44 (17)
Br1—C1—C2—C3	179.81 (14)	C3—C4—C5—C6	0.5 (3)
C1—C2—C3—C4	0.2 (3)	C8—O1—C6—C1	−174.78 (17)
C1—C2—C3—Br2	179.77 (14)	C8—O1—C6—C5	5.2 (3)
C7—O2—C4—C5	5.2 (3)	C2—C1—C6—O1	−179.57 (17)
C7—O2—C4—C3	−175.84 (17)	Br1—C1—C6—O1	0.3 (2)
C2—C3—C4—O2	−179.34 (16)	C2—C1—C6—C5	0.5 (3)
Br2—C3—C4—O2	1.1 (2)	Br1—C1—C6—C5	−179.65 (13)
C2—C3—C4—C5	−0.3 (3)	C4—C5—C6—O1	179.48 (17)
Br2—C3—C4—C5	−179.86 (13)	C4—C5—C6—C1	−0.5 (3)

### Hydrogen-bond geometry (Å, º)

**Table:**

*D*—H···*A*	*D*—H	H···*A*	*D*···*A*	*D*—H···*A*
C7—H7*C*···O1^i^	0.98	2.70	3.632 (3)	160
C1—Br1···*C*_g_^ii^	1.89 (1)	3.75 (1)	5.5701 (19)	161 (1)

Symmetry codes: (i) −*x*, *y*−1/2, −*z*+1/2; (ii) −*x*+1, *y*+1/2, −*z*+1/2.
